# Modification of Fungicide Treatment Needs and Antioxidant Content as a Result of Real-Time Ozonation of Raspberry Plants

**DOI:** 10.3390/molecules29163949

**Published:** 2024-08-21

**Authors:** Natalia Matłok, Tomasz Piechowiak, Małgorzata Szostek, Maciej Kuboń, Pavel Neuberger, Ireneusz Kapusta, Maciej Balawejder

**Affiliations:** 1 Department of Food and Agriculture Production Engineering, University of Rzeszow, St. Zelwerowicza 4, 35-601 Rzeszow, Poland; 2 Department of Chemistry and Food Toxicology, University of Rzeszow, St. Ćwiklińskiej 1a, 35-601 Rzeszow, Poland; tpiechowiak@ur.edu.pl (T.P.); mbalawejder@ur.edu.pl (M.B.); 3 Department of Soil Science Environmental Chemistry and Hydrology, Collegium of Natural Sciences, University of Rzeszów, St. Zelwerowicza 8b, 35-601 Rzeszów, Poland; mszostek@ur.edu.pl; 4 Department of Production Engineering, Logistics and Applied Computer Science, Faculty of Production and Power Engineering, University of Agriculture in Kraków, Balicka 116B, 30-149 Kraków, Poland; maciej.kubon@urk.edu.pl; 5 Faculty of Engineering, Czech University of Life Sciences Prague, Kamycká 129, 165 21 Praha, Czech Republic; neuberger@tf.czu.cz; 6 Department of Food Technology and Human Nutrition, University of Rzeszow, St. Zelwerowicza 4, 35-601 Rzeszow, Poland; ikapusta@ur.edu.pl

**Keywords:** ozone, raspberry plant fumigation, oxidative stress, photosynthesis, phenolic compounds, cyanidin 3-*O*-sophoroside

## Abstract

Raspberry plants need intensive anti-fungal protection. A solution to this problem could be the application of an ozonation process. For this purpose, a technical solution was proposed and implemented in raspberry plant production. The proposal suggests replacing 25% of standard fungicide treatments with ozonation. It was demonstrated that the use of ozone under the proposed conditions made it possible to maintain stable parameters of chlorophyll content and fluorescence (no significant differences), but the intensity of gas exchange was increased. The greatest differences were observed in the second measurement period (T2), when the plants were in the stage of most active development. Additionally, the content and profile of low-molecular-weight antioxidants and the microbial load were determined in the collected fruits. In periods T2 and T3, the proposed method caused a reduction reaching ~2 log cfu g^−1^ in the microbial content of raspberry fruits. It was shown that ozone treatment intensified the biosynthesis of low-molecular-weight antioxidants in fruit (increasing the total polyphenol content by more than 20%). The proposed scheme allows a 25% reduction in standard fungicide treatments while maintaining the health of cultivated raspberry plants. The reduction in fungicide use aligns with the EU regulations and produces fruit with better quality.

## 1. Introduction

Raspberry fruits (*R. idaeus* L.) are globally known and appreciated by consumers for their intense flavour, attractive appearance, and health properties [1]. Fruits from organic cultivation, a process that excludes synthetic pesticides and fertilizers as per EU regulation No. 834/2007 [2], have attracted significant consumer interest. Increasing consumer awareness regarding the quality of consumed raw materials has led to a substantial increase in the sales of certified organic products, including berries. In 2018, the production and consumption of berry fruits rose by 133.3% compared to 2012 [3]. However, meeting the growing demand for raspberry fruits necessitates their production in various systems, including Integrated Pest Management (IPM). Unlike organic production, IPM is characterized by increased production profitability, achieved by reducing the occurrence of numerous pests and pathogenic agents, particularly fungal diseases, leading to increased plant yields [4]. The IPM system for raspberry production is designed to meet both EU regulations on pesticide reduction and the needs of growers, consumers, and supermarkets, while maintaining high fruit quality [5]. Current regulations associated with the European Green Deal, aiming to significantly reduce the use of plant protection products in agriculture and horticulture, drive the search for environmentally friendly methods to support plant protection while ensuring production profitability and the safety and quality of the produced raw materials. Reducing pesticide use in raspberry production, including both field and covered cultivation, is challenging due to the high susceptibility of these fruits to fungal diseases, particularly grey mould [6], which directly affects their quality and durability. Therefore, there is a need to explore innovative, alternative methods for limiting fungal diseases in IPM raspberries while maintaining high fruit quality. It is worth noting that fruits produced in the IPM system often exhibit reduced levels of polyphenols, vitamins, carotenoids, and ellagic acid, as well as malic acid and minerals in the case of berries, compared to fruits from organic cultivation [7]. One potentially promising alternative for limiting fungal pathogens in raspberry cultivation is gaseous ozone. Ozone is an allotropic form of oxygen, characterized by strong disinfectant properties; it effectively combats pathogenic fungi through oxidation, destroying cell membranes, enzymes, and nucleic acids, leading to pathogen deactivation [8]. This is particularly beneficial in protecting crops from serious diseases such as grey mould, powdery mildew, and rot [9]. It should be noted that the application of gaseous ozone as a factor limiting pathogens in plants does not adversely affect plant growth and development, provided that appropriate conditions for the ozonation process are maintained (ozone concentration and process duration). It has been proven that ozonation of plants during their cultivation contributes to maintaining and often improving their physiological parameters. Moreover, ozone has minimal environmental side effects, making it a more sustainable solution than traditional fungicides [10]. The effectiveness of ozone in limiting and combating fungal pathogens has been confirmed in numerous scientific studies [11,12]. However, ozonation technology has mostly been applied to plant materials immediately after harvesting to extend their shelf life by reducing microbial contamination [13] and modifying biosynthesis of bioactive compounds [14,15]. Ozonation of plant material triggers defence mechanisms, resulting in the activation of specific metabolic pathways, which leads to enhanced biosynthesis of bioactive compounds, primarily low-molecular-weight antioxidants [15]. These methods of ozone treatment are primarily conducted in chambers, where plant materials are subjected to the process in batches. The application of the ozone process (fumigation) to raspberry plants in the production process as an alternative method for IPM or organic protection requires the design and implementation of specialized technical solutions that enable continuous and controlled processing. Additionally, before ozone becomes a widely used plant protection method, further research on its optimal concentrations, exposure times, and potential side effects is necessary.

In summary, the use of ozone in eradication of fungal diseases in plants represents an innovative approach, emphasizing effectiveness, environmental safety, and the reduction of traditional chemical pesticide use. As research and development of this technology progress, it may gain increasing recognition as an effective tool in sustainable plant cultivation. The study presented in this paper demonstrates the impact of a cyclical ozone process on the modification of some metabolic pathways (involving low-molecular-weight antioxidants in fruit) and a reduction in fungicide requirements for anti-fungal protection of raspberries grown under cover.

## 2. Results and Discussion

### 2.1. Designed and Constructed Prototype System for Raspberry Plant Fumigation

**Figure 1:**
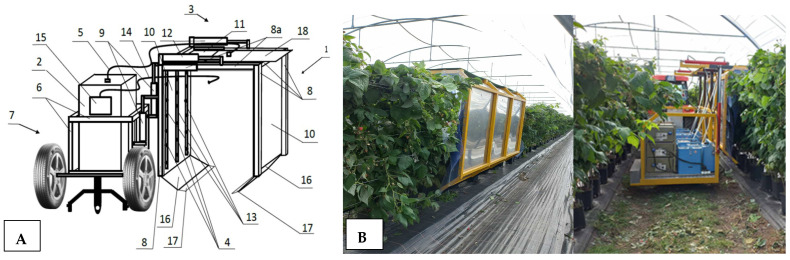
Designed and implemented system for raspberry plant fumigation connected to an agricultural tractor. (**A**) Conceptual diagram, (**B**) Picture of prototype system during O_3_ gas fumigation procedure. NOTE: (1)—fumigation device, (2)—ozone generator, (3)—fumigation chamber, (4)—set of nozzles, (5)—ozone supply hose, (6)—support structure, (7)—mobile carriage, (8)—device frame, (8a)—extendable part of device frame, (9)—vertical actuator, (10)—side walls, (11)—upper wall, (12)—horizontal actuator, (13)—ozone distribution pipe, (14)—suction hose, (15)—ozone concentration sensor, (16)—cover, (17)—comb, (18)—retractable upper cover.

The primary research challenge was to design a device that enables the continuous fumigation of raspberry plants with gaseous ozone under controlled conditions, specifically at experimentally determined exposure times and effective ozone concentrations. Previous solutions demonstrated the effectiveness of ozone treatment, but all ozone experiments were conducted periodically, applying ozone treatment to individual batches of raw materials or plants [16,17]. In real production conditions, such an approach is inefficient, necessitating the design of a mobile device capable of continuously administering the ozone treatment. The device was designed as a suspended platform for a tractor (Figure 1A) (1) or as a self-propelled unit containing an ozone generator (2) and a fumigation chamber (3) equipped with nozzles (4) supplied with ozone through a supply hose (5) from the ozone generator (2). The device is mounted on a mobile carriage (7), on which a device frame (8) is positioned, connected to a support structure (6) and a vertical actuator (9). Two parallel vertical side walls (10) of the fumigation chamber (3) and an upper wall (11) are attached to the frame (8). The frame (8) is designed with flexible connections and extendable elements (8a) allowing the adjustment of the device’s working width and height using a set of actuators (9 and 12). Inside the chamber, there is a set of nozzles (4) arranged on a distribution pipe (13), which runs from the supply hose (5) horizontally along the upper wall (11) and then vertically along the side walls (10), forming a U-shaped line. Inside the fumigation chamber, the end of the suction hose (14) is placed, connected to the concentration sensor (15). The ozone generators are equipped with adjustable oxygen concentrators, enabling control over the quantity of ozone production. By adjusting the device’s movement speed, the raspberry plant row length, and the ozone concentration, the dose of this gas applied to a single plant can be controlled and adjusted. Moreover, the device allows adjustment of the fumigation chamber dimensions (width and height) based on the plant size.

### 2.2. Physiological Parameters of Raspberry Plants

**Figure 2:**
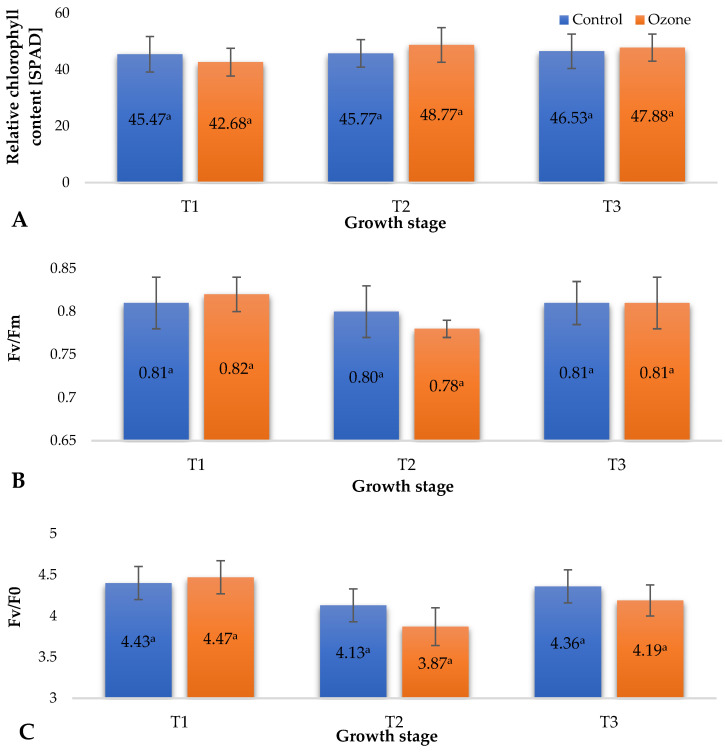
Selected physiological parameters of raspberry plants depending on the applied protection method in three designated periods (beginning of fruiting (T1), full fruiting (T2), and final fruit harvest (T3)). NOTE: Significant differences between the results are indicated by different letters (*n* = 36); significant differences are defined by the criterion *p* < 0.05. (**A**) Relative content of chlorophyll (SPAD). (**B**) Maximal photochemical efficiency of PSII (Fv/Fm). (**C**) Maximum quantum yield of primary photochemistry (Fv/Fo).

Plants’ ability to photosynthesize is directly linked to the amount of chlorophyll [18]. Under various biotic and abiotic stress conditions, such as drought, tissue damage due to pest feeding, or the presence of pathogenic microorganisms, a decrease in chlorophyll content occurs. This phenomenon is often observed in the form of chlorosis, characterized by yellowing of the leaf blade. Reduced chlorophyll content adversely affects the plants’ ability to carry out efficient photosynthesis, leading to restricted growth, development, and fruit yield [19]. Chlorophyll fluorescence parameters, such as Fv/Fm (maximum photochemical efficiency of photosystem II), can serve as indicators not only of photosynthetic efficiency but also of plant health and the presence of stress factors [20]. Figure 2 illustrates the impact of the applied process of fumigating raspberry plants with ozone gas as a supporting measure against pathogenic microorganisms, mainly fungi, on selected physiological parameters of raspberry plants (Figure 2A—Relative content of chlorophyll (SPAD), Figure 2B—Maximal photochemical efficiency of PSII (Fv/Fm), Figure 2C—Maximum quantum yield of primary photochemistry (Fv/Fo)). Analysing the obtained measurement results, no significant changes in the examined parameters were observed after the ozone fumigation treatment of raspberry plants. Ozone, as a strong oxidizing agent, affects the condition of plants, especially during prolonged exposure. This effect, as demonstrated by Rani et al. [21], is mainly associated with the occurrence of photochemical smog, in which plants face prolonged exposure to low concentrations (ppb) of ozone, resulting in impairment of their photosynthetic apparatus. This effect was historically measured through chlorophyll content, but it has been shown that a more reliable method is the measurement of its fluorescence, in which selected parameters indicate the onset of oxidative stress induced by ozone. After prolonged exposure of plants to this gas, plants, especially their green organs, show signs of damage, such as chlorosis and necrosis. In the case of the examined raspberry plants cyclically exposed to short-term (7 s for each plant) application of relatively high concentrations of gaseous ozone (5 ppm), no phytotoxic effects were observed (no necrosis or chlorosis). Recorded results of relative chlorophyll content (SPAD) in raspberry leaves (Figure 2A) showed statistically nonsignificant changes in the content of this pigment in the leaves, which may be due only to the different developmental phases of the plants (between treatment dates) and applied agrotechnical treatments (fertigation, irrigation, etc.). Similar results were noted for the maximal photochemical efficiency of PSII (Fv/Fm) (Figure 2B) and maximum quantum yield of primary photochemistry (Fv/Fo) (Figure 2C). However, in the case of Fv/Fo, greater variations in the values of this parameter were recorded between raspberry plants from the control group (not subjected to O_3_ fumigation) and cyclically ozonated plants compared to the parameter Fv/Fm. It should be emphasized that the Fv/Fo parameter is a more sensitive indicator of plant stress, which directly affects PSII. Due to the lack of correlation between the measured values and carbon assimilation, it allows the detection of stress earlier than in the case of Fv/Fm measurement [22]. The values of Fv/Fo recorded at T2 for raspberry plants fumigated with gaseous O_3_ indicate the occurrence of a stress response. This was the only parameter that indicated any influence of applied ozone on the plants. However, this influence was not phytotoxic. Plants subjected to stressful factors activate a series of defence mechanisms. The action of oxidizing agents, especially reactive oxygen species (ROS), activates enzymes that decompose hydrogen peroxide (CAT) and superoxide anion radicals (SOD). Additionally, other biochemical pathways may be activated (phenylalanine ammonia-lyase (PAL)), triggering increased biosynthesis of selected bioactive compounds from the group of small-molecule antioxidants in various parts of plants, including produced fruit [23]. An increase in the content of these compounds in raw plant materials as a result of exposure to O_3_ can be observed until the onset of phytotoxic effects.

### 2.3. Gas Exchange Parameters

**Figure 3:**
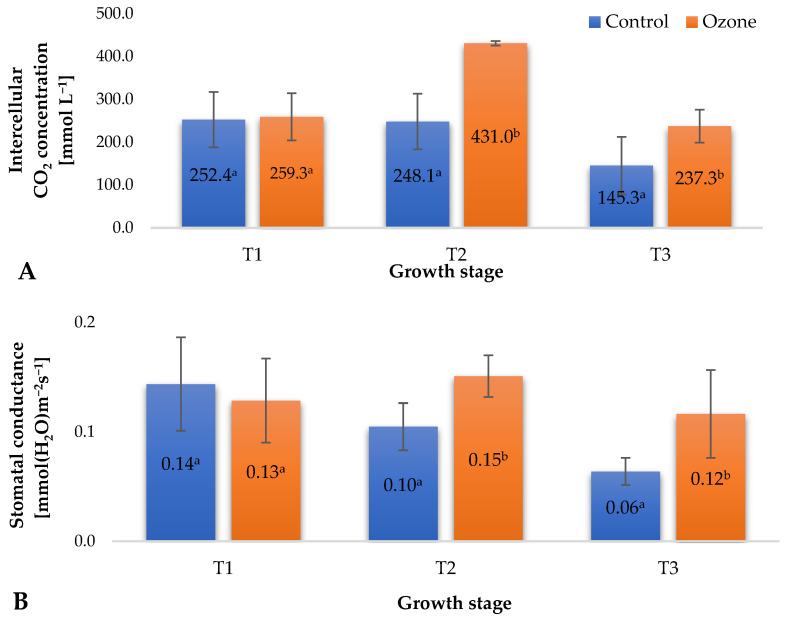

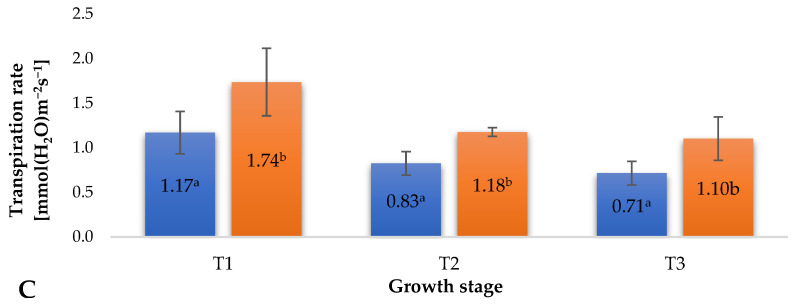
Selected gas exchange parameters in raspberry plants depending on the applied protection method in three designated periods (beginning of fruiting (T1), full fruiting (T2), and final fruit harvest (T3)). NOTE: Significant differences between results are indicated by different letters (*n* = 36); significant differences are defined by the criterion *p* < 0.05. (**A**) Intercellular CO_2_ concentration (Ci). (**B**) Stomatal conductance (gs). (**C**) Transpiration rate (E).

**Figure 4:**
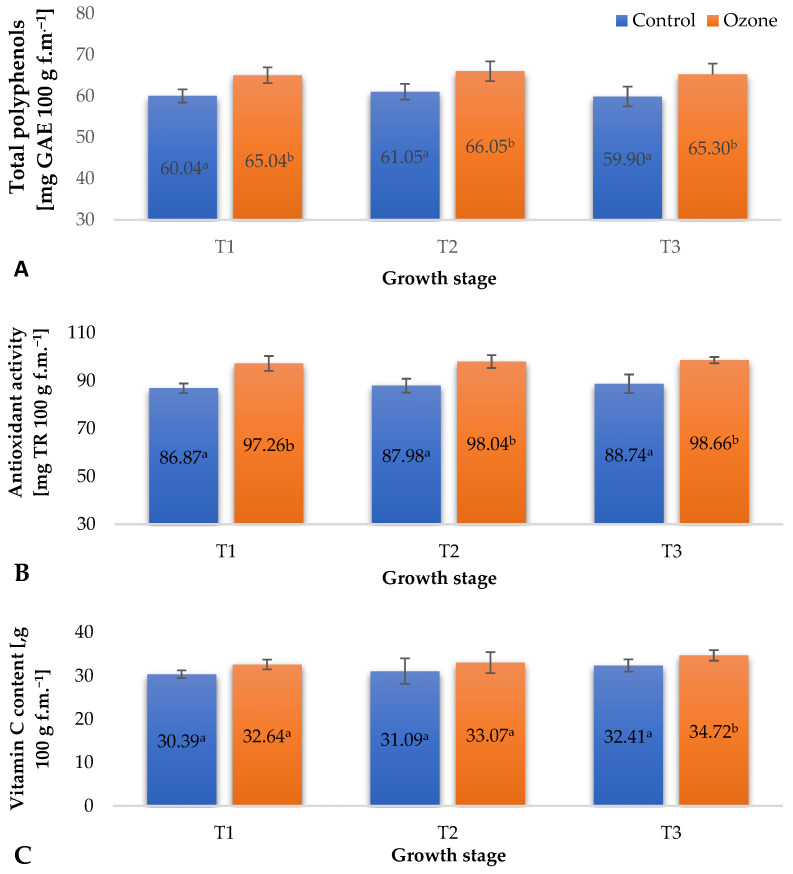
Biochemical parameters of raspberry fruits depending on the applied plant protection method in three designated periods (beginning of fruiting (T1), full fruiting (T2), and final fruit harvest (T3)). NOTE: Significant differences between the results are indicated by different letters (*n* = 9); significant differences are defined by the criterion *p* < 0.05. (**A**) Total polyphenols. (**B**) Antioxidant activity. (**C**) Vitamin C content.

During the cultivation of plants, various abiotic factors influence them, potentially causing stress and disrupting physiological processes, ultimately limiting their growth and yield. Modification of gas exchange is an early response to environmental stress, preceding biomass allocation [24]. The photosynthesis process is particularly sensitive to numerous stress factors, including gaseous ozone, which, upon prolonged exposure, can damage assimilatory tissues [25]. Long-term exposure of plants to ozone leads to leaf drop, with a decrease in the number of stomata remaining. At harmful ozone concentrations, the availability of CO_2_ for plant cells significantly diminishes, leading to a reduction in the photosynthetic rate and consequently limiting plant biomass growth [26]. The treatment applied in the experiment, supporting plant protection against pathogenic agents using gaseous ozone, modified the values of selected gas exchange parameters at designated time points (Figure 3). An increase in the intercellular CO_2_ concentration (Figure 3A) and fluctuations in stomatal conductance (Figure 3B) were observed, seemingly contradicting the limitation of assimilate availability, mainly for CO_2_, due to the applied strategy of ozone treatment in raspberry plants. Stomatal conductance is dependent on the levels of H_2_O_2_ and ethylene generated in plant tissue. The concentrations of both of these factors are also dependent on the presence of ozone in the vicinity of plant tissues [27]. It is essential to note, however, that in some cases, a large accumulation of assimilates in plant leaves can trigger a feedback reaction, potentially leading to photosynthesis inhibition. A negative impact of ozone on plants is reflected in the degree of stomatal opening, which can be estimated by measuring plant transpiration. As suggested by Rai et al. [25], prolonged exposure of plants to ozone, particularly under photochemical smog conditions, can cause partial closure of stomata, leading to reduced transpiration and gas exchange. The treatment supporting raspberry plant protection against pathogenic agents using gaseous ozone significantly increased transpiration (Figure 3C) at all measurement time points. Although this change was significant, no negative effects related to water loss from plant tissues were observed. The recorded gas exchange measurement results (Figure 3) aligned with the determined values of chlorophyll fluorescence parameters (Figure 2) and indicated the good condition of the plants, with no adverse effects from the applied ozone treatment strategy. The noted differences between the ozone-treated samples and the control group suggest the occurrence of mild, controlled oxidative stress, corroborated by the recorded increases in the levels of small-molecule antioxidants in the fruits (Figure 4).

### 2.4. Qualitative Parameters of Fruits

#### 2.4.1. Content of Bioactive Compounds

The content of bioactive compounds in fruits, especially those from the group of low-molecular-weight antioxidants, can be shaped by various factors modifying their metabolism. It has been demonstrated that one such factor can be ozone, which, through the activation of selected enzymatic systems, induces enhanced biosynthesis of phenolic compounds, vitamin C, and other compounds shaping the total antioxidant potential. It is worth noting that this modification using O_3_ can be carried out at the cultivation stage as well as directly after the harvest of raw plant materials (including fruits), when they retain the characteristics of living organisms (perform gas exchange and other physiological processes) [28]. The recorded results of physiological measurements of raspberry plants, especially the maximum quantum yield of primary photochemistry (Fv/Fo), indicated the occurrence of a stress reaction induced in raspberry plants by cyclic fumigation with gaseous O_3_ (Figure 2C). In many cases, these factors induce metabolic changes in plants, the result of which is the enhanced biosynthesis of molecules capable of neutralizing the adverse effects of ozone. In the case of applying the ozone process to fruits that have retained the ability to metabolize, the activation of metabolic pathways allowing the generation of increased amounts of selected antioxidant compounds is observed. It seems that this mechanism is replicated when applying the ozone process to fruit-bearing plants. In all measurement periods, an increase in the total content of polyphenols (Figure 4A), the antioxidant potential (Figure 4B), and the vitamin C content (Figure 4C) of raspberries from ozonated plants was noted. However, these increases were not as significant as in the case of fruits ozonated after harvest, which directly results from both the applied concentration of gaseous ozone, which, in the case of post-harvest ozone treatment, is usually several tens of ppm, and the exposure time to the action of this gas, which can be up to 30 min [29]. The recorded levels of bioactive compounds in raspberries harvested from ozonated plants confirm that the developed method of supporting plant protection using gaseous ozone can not only reduce the use of pesticides (fungicides) in raspberry cultivation, aligning with the European policy and implementation of The European Green Deal, but also contribute to the production of fruits with an increased content of selected bioactive compounds. One of the most important parameters defining the agricultural technology of raspberry cultivation is fruit yield. In the control sample and in the plants subjected to treatment, the collected fruit was weighed to determine the yield. From the plants subjected to treatment, 2.32 ± 0.18 kg plant^−1^ was collected, while the control plants yielded 2.30 ± 0.18 kg plant^−1^.

#### 2.4.2. Profile of Polyphenolic Compounds

Raspberry fruits are a valuable source of phenolic compounds, the composition of which is a characteristic feature and is usually consistent for a given chemotype. As shown in Figure 4A, the use of a treatment to support the growth and development of plants with gaseous ozone fumigation altered the total content of phenolic compounds in the harvested fruits. Due to the lack of selectivity of the utilized Folin–Ciocalteu method, fruits collected at time point T3 were analysed to determine the phenolic compound profile (Table 1). The data from MS made it possible to calculate the content of each compound. The percentage composition of the mixture was determined using the UPLC-MS method, and the total content of phenolic compounds using the Folin method. This analysis aimed to determine whether the plants exposed to gaseous ozone fumigation merely intensified their production of this group of compounds in the produced fruits or whether selected metabolic pathways leading to the biosynthesis of specific phenolic compounds were modified. The analysis of the obtained results indicates that the differences observed were solely due to the standard error of the applied analytical method. The main phenolic compound identified in the fruits collected from both ozonated and control raspberry plants was cyanidin 3-*O*-sophoroside. The remaining analysed compounds were at the same level, as confirmed by the statistical analysis. The only difference was observed in the content of Cyanidin 3-O-glucoside, which is presumably formed by enhanced hydrolysis of one glycoside bond in cyanidin 3-O-sophoroside. Presumably, the increased biosynthesis of these compounds was associated with the non-selective activation of biochemical systems generating phenolic compounds. This is a typical effect of using ozone as an abiotic elicitor [30]. As demonstrated in the work of Sachadyn-Król et al. [31], the activation of phenylalanine ammonia-lyase (PAL) by gaseous ozone results in enhanced biosynthesis of phenolic compounds with a qualitative composition characteristic of the given plant. 

### 2.5. Microbiological Quality of Fruits

The effectiveness of the proposed method for supporting the protection of raspberry plants using ozone gas fumigation with the designed system was evaluated not only through cyclic observations of the extent of plant infection by grey mould, raspberry anthracnose, and shoot dieback but also through microbiological analyses of the fruits. It is important to note that in all observation periods (every 5 days during plant vegetation), no signs of plant infection by these fungal pathogens were observed in either the control variant or the experimental variant. The absence of visible symptoms of plant infection by fungal pathogens does not guarantee the absence of their spores in fruits, which may be damaged during storage due to their multiplication. To assess the impact of the applied method for supporting raspberry plant cultivation on the microbiological load of harvested fruits, analyses were conducted to evaluate the number of colony-forming units of yeasts and moulds, mesophilic lactic acid fermentation bacteria, mesophilic aerobic bacteria, and anaerobic spore-forming bacteria (Table 2). The applied protection scheme (control variant) and support using ozone gas (experimental variant) eliminated the presence of most bacterial pathogens on the fruits but were not fully effective against yeasts and moulds, which are the main cause of storage diseases directly affecting the durability and safety of these fruits. However, a significant impact of ozone fumigation on plants was observed in reducing the microbiological load of yeasts and moulds on fruits. In period T1, this reduction was approximately ~1 log cfu g^−1^, while in the remaining periods (T2 and T3), this method proved even more effective, with a reduction reaching ~2 log cfu g^−1^. The industrial application of ozone is mainly directed towards reducing the presence of microorganisms and decomposing organic substances polluting various matrices, mainly drinking water. In these cases, it has been shown that ozone is much more effective than other factors such as chlorine or ethylene oxide and decomposes into molecular oxygen during activity without leaving residues. In many cases, post-harvest ozone treatment of fruits has demonstrated not only antimicrobial effectiveness but also multi-directional mechanisms enabling the extension of the shelf life. One such aspect, especially for climacteric fruits, is the impact of ozone on inhibiting the expression of S-adenosyl-L-methionine SAM, which breaks down into SAM, a precursor to ethylene [32].

## 3. Materials and Methods

### 3.1. Research Material and Experimental Design

In the experiment, two-year-old raspberry plants of the variety Malling Bella were used, cultivated soillessly in 10 L production pots under cover (in a greenhouse). The pots with plants were arranged in rows with a distance of approximately 30 cm between them. The length of a single row was 200 m. The control variant consisted of a greenhouse containing 8 rows, where chemical protection against fungal diseases was carried out using pesticides approved for raspberry cultivation. In this variant, a total of 12 treatments were applied during the vegetation period using the following fungicides: Switch 62.5 WG (Syngenta, Wilmington, DE, USA) and Captan 50WP (Drexel Chemical Company, Memphis, TN, USA). The experimental variant was a similar setup, but the number of fungicide treatments was reduced by 25% (9 treatments) through the application of ozone treatment using a designed fumigation system. The ozone treatment was alternately performed in place of every fourth fungicide treatment. Plant protection with gaseous ozone commenced synchronously with the first conventional (fungicidal) protection treatment and continued throughout the entire vegetation period until the fruit harvest. Coconut fibre served as the substrate for raspberry cultivation. Automated fertigation was carried out during plant growth, providing standard macro- and micronutrient solutions. The raspberry plant fumigation process was conducted with a constant device movement speed of 2 km h^−1^ (lowest speed of tractor) and an ozone concentration of 5 ppm. The concentration was determinate by the ozone-generating capacity of the Korona L 400 TOWER ozone generator in the chamber (Korona Scientific and Implementation Laboratory, Piotrków Trybunalski, Poland). The exposure time of each individual plant to ozone was 7 s, determined by the chamber length (3.9 m) and the applied speed. These conditions made it possible to conduct the plant fumigation process with gaseous ozone below the phytotoxicity level. The ozone treatment process utilized Korona L 400 TOWER ozone generators equipped with Philips Everflo OPI/IKK oxygen concentrators (Respironics, Murrysville, PA, USA). The ozone concentration in the fumigation chamber was measured using the 106 M ozone detector by 2B Technologies (Ozone Solution, Hull, MA, USA). The impact of the applied plant protection process using the designed fumigation system was assessed by applying analytical methods, measuring physiological parameters of plants at selected intervals, and analysing selected fruit parameters. Additionally, plant disease observations, particularly on fungal diseases, were conducted cyclically (every 5 days) throughout the raspberry plant growth period. Based on these observations, no evidence of plant infection with fungal diseases, including grey mould, raspberry anthracnose, and shoot dieback, was found in either the control or the experimental variant.

### 3.2. Measurement of Physiological Parameters of Plants

The impact of the applied plant protection regimen designed to support raspberry plants against fungal diseases was assessed by measuring the physiological state of the plants through relative chlorophyll content in leaves, chlorophyll fluorescence parameters, and parameters defining photosynthetic intensity. Measurements were conducted at three designated time points: the beginning of fruiting (T1), full fruiting (T2), and the final fruit harvest (T3). The measurement of relative chlorophyll content in raspberry leaves was carried out using the leaf greenness index method (SPAD) with the SPAD 502 device (Konica-Minolta Inc., Osaka, Japan), following the methodology described by Matłok et al. [14]. Selected chlorophyll fluorescence parameters, including maximum quantum efficiency of PSII photochemistry (Fv/Fm) and maximum quantum efficiency of primary photochemistry (Fv/F0), were measured using a fluorimeter analyser (Pocket PEA, Hansatech Instruments, King’s Lynn, Norfolk, UK), following the methodology described by Jańczak-Pieniążek et al. [33]. Additionally, using the portable LCpro-SD photosynthesis measurement system (ADC BioScientific Ltd., Hoddesdon, UK), net photosynthesis rates (PN), transpiration rates (E), stomatal conductance (gs), and intercellular CO_2_ concentration (Ci) were determined. Measurements were conducted on fully expanded leaves located on one-year-old shoots. The measurements were performed in 36 replicates for each experimental variant.

### 3.3. Determination of Bioactive Compounds Content in Fruits

The impact of gaseous ozone plant fumigation on the quality of produced raspberry fruits was assessed by determining the total polyphenol content using the Folin–Ciocalteu method, following the methodology described by Piechowiak et al. [32] with minor modifications. A sample was prepared by homogenizing 5 g of frozen (−67 °C) and ground fruits with 15 mL of 75% methanol. After centrifugation at 10,000× *g* for 30 min (4 °C), the supernatant was used for analysis. The total polyphenol content was expressed as gallic acid equivalents. In the same supernatant, the antioxidant activity against DPPH radicals was also determined, following the method presented by Piechowiak et al. [34]. The results were expressed as Trolox equivalents. The vitamin C content was determined by spectrophotometric method using DCPiP reagent, following Piechowiak et al. [34]. For vitamin C extraction, 5 g of frozen tissue was homogenized with 15 mL of 2% oxalic acid. In the obtained homogenates, the profile of phenolic compounds was determined using the UPLC-PDA-MS/MS method according to the protocol proposed by Balawejder et al. [35]. 

### 3.4. Microbiological Analysis of Fruits

Raspberry fruits collected from the experimental variants were subjected to microbiological analysis to determine the impact of the applied plant protection method on the colony-forming units of yeast and moulds, mesophilic lactic acid fermentation bacteria, mesophilic aerobic bacteria, and anaerobic spore-forming bacteria. The analyses were performed according to the methodologies described in Matłok et al. [14].

### 3.5. Statistical Analysis

The obtained results were analysed using STATISTICA 13.1 software (StatSoft, Palo Alto, CA, USA). One-way analysis of variance (ANOVA) and a post hoc Tukey HSD test were applied (α = 0.05) to show the differences between the impact of the plant protection method used on selected gas exchange parameters, relative chlorophyll content, chlorophyll fluorescence, and selected bioactive compounds in fruit.

## 4. Conclusions

During this research, a new method was developed for supporting plants against fungal diseases using ozone fumigation with an innovative device designed for this purpose, which is compatible with agricultural tractors. This device enables controlled fumigation (allowing plants to be dosed with ozone at a specified concentration and exposure time) in a continuous system. The proposed scheme allows a 25% reduction in standard fungicide treatments while maintaining the health of cultivated raspberry plants. It should be noted that the research was conducted under conditions close to real-life scenarios, with the replacement of standard fungicides aimed at reducing the risk of diseases caused by the lack of standard planned protection. Commercial production was carried out on the plantation despite the research being conducted. Additionally, changes in the metabolic pathways of small antioxidant molecules in the produced fruits were observed. Increased biosynthesis of these substances in fruits was observed, with no change in their qualitative composition. This enables the production of fruits with enhanced quality parameters. Furthermore, it was demonstrated that fruits harvested from plants subjected to ozone fumigation exhibited significantly lower microbiological loads than conventionally produced fruits (the control sample). This significantly affects not only their quality but also their storage durability, which is crucial for raspberry fruits.

## Figures and Tables

**Table 1:** Individual phenolic compounds identified by UPLC-PDA-MS/MS in raspberry fruits depending on ozone fumigation.

No	Compound	RT	[M − H]^+/−^	Fragment Ions	Absorbance Maxima	Content [%]	
		(min.)	(*m*/*z*)	(*m*/*z*)	(nm)	Control	Ozone
**1**	Caffeic acid glucoside	2.58	341^−^	179	299sh. 324	1.21 ^a^ ± 0.11	1.04 ^a^ ± 0.09
**2**	Cyanidin 3-*O*-sophoroside	2.77	611^+^	287	279. 509	68.87 ^a^ ± 2.48	68.34 ^a^ ± 4.11
**3**	Cyanidin 3-*O*-glucosyl-rutinoside	2.91	757^+^	611. 287	279. 517	13.88 ^a^ ± 0.41	13.45 ^a^ ± 2.87
**4**	Cyanidin 3-*O*-glucoside	3.04	449^+^	287	279. 515	4.71 ^a^ ± 0.08	6.21 ^b^ ± 0.10
**5**	Cyanidin 3-*O*-rutinoside	3.18	595^+^	287	278. 512	4.35 ^a^ ± 0.38	4.78 ^a^ ± 2.08
**6**	Procyanidin dimer type B	3.23	577^−^	289	274	1.13 ^a^ ± 0.04	0.98 ^a^ ± 0.06
**7**	(+)Catechin	3.69	289^−^	144	274	1.55 ^a^ ± 0.21	1.28 ^a^ ± 0.13
**8**	Ellagic acid rhamnoside	3.97	447^−^	301	360	1.08 ^a^ ± 0.19	1.03 ^a^ ± 0.17
**9**	Casuarinin	4.11	935^−^	633. 301	244	0.78 ^a^ ± 0.03	0.72 ^a^ ± 0.05
**10**	Lambertianin C	4.20	1401^−^	633. 301	244	0.89 ^a^ ± 0.04	0.73 ^a^ ± 0.04
**11**	Ellagic acid pentoside	4.40	433^−^	301	360	0.73 ^a^ ± 0.07	0.68 ^a^ ± 0.02
**12**	Quercetin 3-*O*-rhamnoside	5.55	447^−^	301	255. 350	0.83 ^a^ ± 0.12	0.76 ^a^ ± 0.09

NOTE: Significant differences between the results are indicated by different letters. Significant differences are defined by the criterion *p* < 0.05.

**Table 2:** Microbiological load of raspberry fruits depending on the applied plant protection method. NOTE: Significant differences between the results are indicated by different letters (*n* = 12); significant differences are defined by the criterion *p* < 0.05.

Number of Treatment	Treatment Variant	Count of Mesophilic Lactic Acid Bacteria [cfu g^−1^]	Presence of Anaerobic Spore Bacteria [cfu g^−1^]	Count of Aerobic Bacteria [cfu g^−1^]	Count of Yeast and Mould [cfu g^−1^]
T1	control	<1.0 × 10^1^	absence	<1.0 × 10^1 a^	4.8 × 10^4^ ± 2.1 × 10^5 b^
	ozone	<1.0 × 10^1^	absence	<1.0 × 10^1 a^	5.1 × 10^3^ ± 3.6 × 10^2 a^
T2	control	<1.0 × 10^1^	absence	<1.0 × 10^1 a^	3.9 × 10^5^ ± 7.3 × 10^3 b^
	ozone	<1.0 × 10^1^	absence	<1.0 × 10^1 a^	4.3 × 10^3^ ± 3.7 × 10^2 a^
T3	control	<1.0 × 10^1^	absence	<1.0 × 10^1 a^	6.3 × 10^5^ ± 5.8 × 10^4 b^
	ozone	<1.0 × 10^1^	absence	<1.0 × 10^1 a^	5.9 × 10^3^ ± 3.7 × 10^2 a^

## Data Availability

The original contributions presented in the study are included in the article, further inquiries can be directed to the corresponding author.
